# RETRACTED: Ahmad et al. Phytomedicine-Based Potent Antioxidant, Fisetin Protects CNS-Insult LPS-Induced Oxidative Stress-Mediated Neurodegeneration and Memory Impairment. *J. Clin. Med.* 2019, *8*, 850

**DOI:** 10.3390/jcm15176785

**Published:** 2026-09-01

**Authors:** Ashfaq Ahmad, Tahir Ali, Shafiq Ur Rehman, Myeong Ok Kim

**Affiliations:** Division of Applied Life Science (BK 21), College of Natural Sciences, Gyeongsang National University, Jinju 52828, Republic of Korea; ash_phr@yahoo.com (A.A.); tahirneuro@gmail.com (T.A.); shafiq.qau.edu@gmail.com (S.U.R.)

The Journal retracts the article “Phytomedicine-Based Potent Antioxidant, Fisetin Protects CNS-Insult LPS-Induced Oxidative Stress-Mediated Neurodegeneration and Memory Impairment” [1] cited above.

Following publication, concerns were brought to the attention of the Editorial Office regarding the integrity of the figures presented in this article [1].

In accordance with the journal’s standard procedures, the Editorial Office and Editorial Board conducted an investigation, which identified indicators of inappropriate editing and duplication in Figures 5 and 6. Although the authors cooperated with the investigation, the explanations and supporting materials provided were not deemed sufficient by the Editorial Board to resolve the identified concerns.

As a consequence, the Editorial Board has lost confidence in the integrity of the images and the reliability of the findings and has decided to retract this publication in accordance with MDPI’s retraction policy.

This retraction was approved by the Editor-in-Chief of the *Journal of Clinical Medicine*.

The authors have been informed of this retraction and have indicated their disagreement with this decision.
